# Secreted frizzled-related protein 2: a key player in noncanonical Wnt signaling and tumor angiogenesis

**DOI:** 10.1007/s10555-020-09941-3

**Published:** 2020-11-02

**Authors:** Karlijn van Loon, Elisabeth J. M. Huijbers, Arjan W. Griffioen

**Affiliations:** Angiogenesis Laboratory, Cancer Center Amsterdam, Department of Medical Oncology, VU University Medical Center, Amsterdam UMC, Amsterdam, The Netherlands

**Keywords:** Angiogenesis, SFRP2, Wnt signaling, Cancer, Therapeutic target, Tumor vasculature

## Abstract

Secreted frizzled-related proteins (SFRP) are glycoproteins containing a so-called frizzled-like cysteine-rich domain. This domain enables them to bind to Wnt ligands or frizzled (FzD) receptors, making potent regulators of Wnt signaling. As Wnt signaling is often altered in cancer, it is not surprising that Wnt regulators such as SFRP proteins are often differentially expressed in the tumor microenvironment, both in a metastatic and non-metastatic setting. Indeed, SFRP2 is shown to be specifically upregulated in the tumor vasculature of several types of cancer. Several studies investigated the functional role of SFRP2 in the tumor vasculature, showing that SFRP2 binds to FzD receptors on the surface of tumor endothelial cells. This activates downstream Wnt signaling and which is, thereby, stimulating angiogenesis. Interestingly, not the well-known canonical Wnt signaling pathway, but the noncanonical Wnt/Ca^2+^ pathway seems to be a key player in this event. In tumor models, the pro-angiogenic effect of SFRP2 could be counteracted by antibodies targeting SFRP2, without the occurrence of toxicity. Since tumor angiogenesis is an important process in tumorigenesis and metastasis formation, specific tumor endothelial markers such as SFRP2 show great promise as targets for anti-cancer therapies. This review discusses the role of SFRP2 in noncanonical Wnt signaling and tumor angiogenesis, and highlights its potential as anti-angiogenic therapeutic target in cancer.

## Introduction

**Fig. 1:**
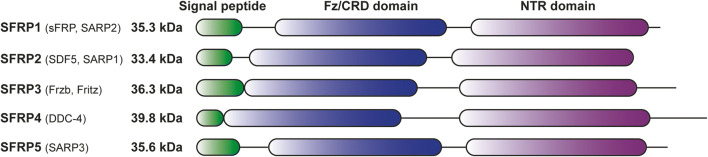
The SFRP family members contain a signal peptide (green), frizzled-like cysteine-rich domain (Fz/CRD; blue), and netrin domain (NTR; purple). Synonyms for each protein are indicated between brackets

The secreted frizzled-related protein (SFRP) family consists of five secreted glycoproteins: SFRP1, SFRP2, SFRP3, SFRP4, and SFRP5 (Fig. 1). From a phylogenetic perspective, SFRP1, SFPR2, and SFRP5 form an SFRP subfamily based on their sequence similarities [1]. All five family members contain a signal peptide, a netrin domain (NTR), and a frizzled-like cysteine-rich domain (Fz/CRD) (Fig. 1). The signal peptide is important in the secretion process of SFRP2 and is likely to be absent in the mature secreted protein. The C-terminal NTR domain contains six conserved cysteine residues, able to form a total of three disulfide bridges. This domain shows homology to the netrin domain found in complement proteins C3, C4, C5, type I procollagen C-proteinase enhancer proteins, and tissue inhibitors of metalloproteinases [2]. The Fz/CRD domain present in SFRP proteins is highly similar to the extracellular Wnt binding domain of FzD receptors [3], enabling binding between SFRP proteins to Wnt ligands. The SFRP family is known to be involved in the regulation of Wingless-related integration site (Wnt) signaling, an important pathway not only in embryonic development, tissue regeneration, and cell proliferation, but also in carcinogenesis [4]. This pathway is activated by binding of soluble Wnt ligands to frizzled (FzD) receptors on the cell surface, and eventually leads to the transcription of Wnt target genes.

Although overactivation of the Wnt signaling pathway is inextricably linked to cancer initiation and progression, it also plays an important role in the tumor vasculature. Interestingly, SFRP2 is described to be overexpressed in the tumor vasculature of breast cancer tissues [5]. In addition, SFRP2-directed ultrasound imaging clearly shows specific signal in the tumor vasculature, while normal vessels are not visualized [6]. Proteins such as SFRP2, that seem to be specifically (over)expressed in the tumor vasculature, show great promise as therapeutic targets in the fight against cancer [7–9]. To enhance insight in oncogenic role of SFRP2, we performed a literature study about SFRP2 in Wnt signaling and tumor angiogenesis.

## SFRP2 and Wnt signaling

More than four decades ago, studies on mutagenesis of the W*ingless* gene in *Drosophila melanogaster* showed development defects in the wings of the fruit fly [10, 11]. A few years later, a new oncogene *Int1* was identified to be involved in mouse mammary tumor formation [12]. Subsequently, it was discovered that the highly conserved *Int1* was already known as W*ingless*, and is therefore currently referred to as Wnt1 [13]. Twenty-three years after the discovery of *Wingless*, SFRP2 was discovered in a cDNA screen for secreted and transmembrane proteins in bone marrow stromal cells [14]. In this screen, Shirozu et al. identified a new protein which was named stromal cell–derived factor (SDF)-5. The C-terminal end of SDF-5 showed high similarity to the *Frizzled* gene of *Drosophila*, and the protein was therefore later renamed an SFRP. One year later, Melkonyan et al. discovered the presence of an anti-apoptotic protein in the culture medium of quiescent mouse embryonic cells [15]. This protein was initially named secreted apoptosis-related protein (SARP)-1, but it turned out to be the same protein, which is now referred to as SFRP2. The human *SFRP2* gene is located on chromosome 4q31.3 and encodes a 295-aa protein [16].

Members of the SFRP family were initially described to be antagonists of Wnt signaling, due to their sequestration of Wnt ligands, which prevents binding of Wnt ligands to FzD receptors. However, many researchers have proposed an additional agonistic effect on Wnt signaling by direct binding to FzD receptors or by influencing the Wnt activating effect of soluble Wnt ligands.

### SFRP2 and canonical Wnt signaling

**Fig. 2:**
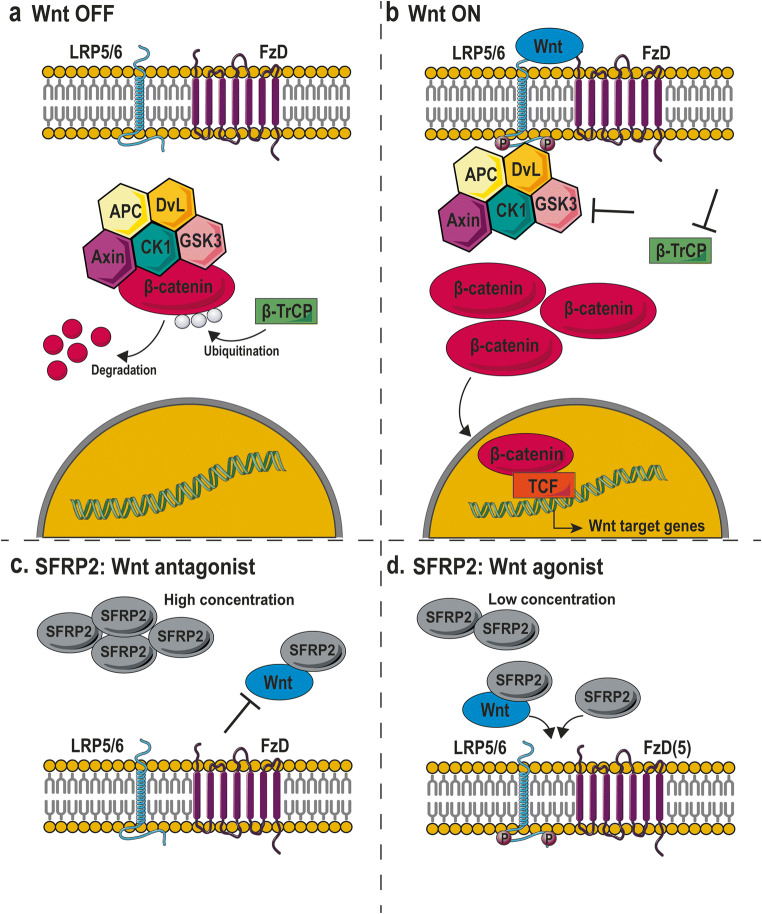
SFRP2 and the canonical Wnt signaling pathway. **a** In the absence of Wnt ligands, an activated destruction complex consisting of proteins such as DvL and APC enables β-catenin ubiquitination by β-TrCP and subsequent degradation of β-catenin in the proteasome. **b** When Wnt ligands binds to FzD receptors, LRP becomes phosphorylated and the destruction complex moves towards the plasma membrane. By this means, the destruction complex remains inactive and, therefore, β-catenin is not ubiquitinated and degraded. Finally, β-catenin translocates to the nucleus and transcription of Wnt target genes is initiated. **c** Several studies suggest that the effect of SFRP2 on Wnt signaling is dependent on the concentration. High concentrations are described to drive towards Wnt antagonism. SFRP2 is able to sequester Wnt ligands and prevent binding to FzD receptors. **d** At low concentrations of SFRP2, SFRP2 is able to synergize with Wnt ligands, enhancing their ability to activate Wnt signaling. Interestingly, SFRP2 is also described to directly bind to FzD receptors, activating the signaling cascade. The agonistic effect of SFRP2 might also be context dependent, where the expression of FzD5 is crucial for SFRP2 to function as potent Wnt activator. This figure was prepared using Servier Medical Art under a Creative Commons Attribution 3.0 Unported License

The canonical Wnt signaling pathway, also known as the Wnt/β-catenin pathway, plays an important role in embryogenesis, cell growth, and proliferation [10]. In short, in the absence of Wnt ligand binding, a destruction complex is present in an active formation in the cytoplasm (Fig. 2). This destruction complex consists of several proteins including the dishevelled protein (DvL), Axin, and adenomatous polyposis coli (APC), and is responsible for the degradation of β-catenin in the absence of Wnt ligand binding. β-catenin degradation is initiated by its ubiquitination by the enzyme F-box β-transducing repeat-containing protein (β-TrCp), which eventually results in proteasomal degradation of β-catenin (Fig. 2a).

Several Wnt ligands are able to induce canonical Wnt signaling, such as Wnt3a, Wnt4, Wnt6, Wnt9A, and Wnt10B [17]. In the situation where Wnt ligands bind to FzD receptors, the low-density lipoprotein receptor–related proteins (LRP) become phosphorylated (Fig. 2b). This eventually leads to a translocation of the destruction complex towards the FzD receptor near the cell membrane. Consequently, DvL can bind to LRP and becomes activated, preventing the activation of the destruction complex. This means that the β-TrCp is not able to ubiquitinate β-catenin, and β-catenin is consequently not degraded in the proteasome. Therefore, β-catenin can translocate to the nucleus and induce transcription of target genes, involved in a variety of processes such as proliferation, differentiation, migration, and apoptosis (Fig. 2b) [10, 18].

SFRP2 can function as Wnt antagonist, indicated by the fact that mesenchymal stem cells overexpressing SFRP2 show decreased Wnt activity, observed by lower levels of β-catenin [19]. The most likely mechanism of Wnt antagonism is the sequestering of soluble Wnt ligands, preventing their binding to FzD receptors. Wawrzak et al. have shown that both SFRP1 and SFRP2 are able to bind Wnt3a, a Wnt ligand able to activate the canonical Wnt signaling pathway [20]. This data further confirms the potential suppressive role of SFRP2. Direct binding between SFRP2 and Wnt3a was also confirmed by Hua et al. [21]; this binding is mediated *via* the Fz/CRD domain of SFRP2. Exposing enteroendocrine L cells to both Wnt3a and SFRP2 results in a potent inhibition of Wnt3a activity [20, 22]. While SFPR2 alone did not affect the accumulation of β-catenin, combining SFRP2 with Wnt3a diminished the accumulation observed in single treatment with Wnt3a [20]. Since Wnt3a is an activator of the canonical Wnt signaling pathway, several papers reported the effect on proliferation after Wnt3a and SFRP2 exposure. When Wnt3a expression was introduced in the neural tube of chick embryos, a significant increase in proliferation was observed compared to embryos that were not electroporated with the *Wnt3a* gene [22]. In contrast, when both Wnt3a and SFRP2 were co-expressed, no effect on proliferation was observed. The same inhibitory effect on Wnt signaling is described for Wnt1, Wnt4, and Wnt9a in COS7, a fibroblast cell line derived from monkey kidneys [23]. These data indicate that SFRP2 is able to inhibit Wnt3a-mediated canonical Wnt signaling *in vitro* and *in vivo*, likely by sequestering Wnt ligands and inhibiting their binding to FzD receptors (Fig. 2c).

Since Wnt3a was also shown to be upregulated in response to hypoxia, Zhang et al. tested the effect of SFRP2 on Wnt3a activity in H9C2 cells under hypoxia [24]. Incubation of these rat embryonic heart-derived myoblasts with recombinant Wnt3a resulted in increased caspase activity, demonstrating the apoptotic effect of Wnt3a. Both the caspase activity and the nuclear β-catenin levels were greatly reduced in the presence of SFRP2. These data indicate that the anti-apoptotic function of SFRP2 might, at least partly, be explained by inhibition of the canonical Wnt signaling pathway.

Besides its well-known role as Wnt signaling antagonist, several studies propose an agonistic effect of SFRP2 on the Wnt signaling pathway (Fig. 2d). In primary cultures of intestinal epithelium, the presence of SFPR2 clearly induced the expression of the cell cycle regulators *C-myc* and *cyclin D1*, and consequently cell proliferation [25]. The same effect was observed for primary cultures exposed to SFRP2 and Wnt3a, or Wnt3a alone. These data suggest that SFRP2 is a positive regulator of Wnt signaling, likely by direct binding to FzD receptors (Fig. 2d). To further confirm the agonistic role of SFRP2, Mastri et al. treated cardiac fibroblasts with recombinant SFRP2, in the presence or absence of an anti-SFPR2 antibody [26]. While recombinant SFRP2 strongly enhanced *Axin2* and *Wnt3a* gene expression and nuclear β-catenin accumulation, the addition of anti-SFPR2 antibodies abrogated this effect. These antibodies reduced apoptosis and enhanced angiogenesis after myocardial infarction [26, 27]. Activation of Wnt signaling by SFRP2 is also observed in endometriosis, in which SFRP2 is highly upregulated compared to normal endometrium [28]. When SFRP2 expression was diminished in primary cultured extraovarian endometriotic cells, β-catenin levels were significantly reduced compared to control cells. Furthermore, SFRP2 can also enhance Wnt signaling by potentiating the Wnt activating effect of Wnt3a (Fig. 2d). Treatment of HEK293 human embryonic kidney cells with both SFRP2 and Wnt3a significantly increased β-catenin accumulation and LRP6 receptor phosphorylation compared to treatment with Wnt3a alone [21, 29]. Importantly, recombinant SFRP2 alone did not affect the amount of β-catenin in this embryonic kidney cell line. This agonistic activity was also confirmed in other cells such as C2C12 murine myoblasts [21].

In order to learn more about the functional role of SFRP2 in canonical Wnt signaling, several different *Sfrp2*^−/−^ mice were generated. When comparing activated β-catenin levels in the intestine of WT or *Sfrp2*^−/−^ mutant mice, lack of SFRP2 results in reduced Wnt activity [30]. This agonistic effect of SFRP2 was further confirmed *in vitro*, whereby transfection of COS7 cells with a low concentration of SFRP2 strongly activated Wnt signaling. Interestingly, transfection with a high dose of SFRP2 resulted in the inhibition of Wnt signaling [30]. This suggests that the antagonistic or agonistic effect of SFRP2 might depend on the expression level (Fig. 2c, d). Xavier et al. further investigated SFRP2 as a double-edged sword in canonical Wnt signaling [31]. Indeed, they confirmed the hypothesis regarding its concentration-dependent effect: treatment of mouse mammary epithelial cells and L cells with a low concentration of SFRP2 resulted in a strong increase in signaling compared to Wnt3a alone, an effect that was heavily reduced at a high SFRP2 concentration. However, when they repeated the same experiment using HEK293/STF cells—kidney cells that are adapted to express luciferase upon canonical Wnt signaling—all tested concentrations of SFRP2 were able to enhance Wnt signaling [31]. They propose that the concentration-dependent effects observed in different cellular contexts might be explained by differences in Wnt receptor expression. Indeed, when L cells were molecularly adapted to express FzD5, a receptor usually not expressed on parental L cells, all concentrations were able to increase the Wnt3a signaling pathway (Fig. 2d). However, further research is needed to investigate the exact mechanisms behind the concentration- and context-dependent effects of SFRP2 on Wnt signaling.

### SFRP2 and noncanonical Wnt signaling

**Fig. 3:**
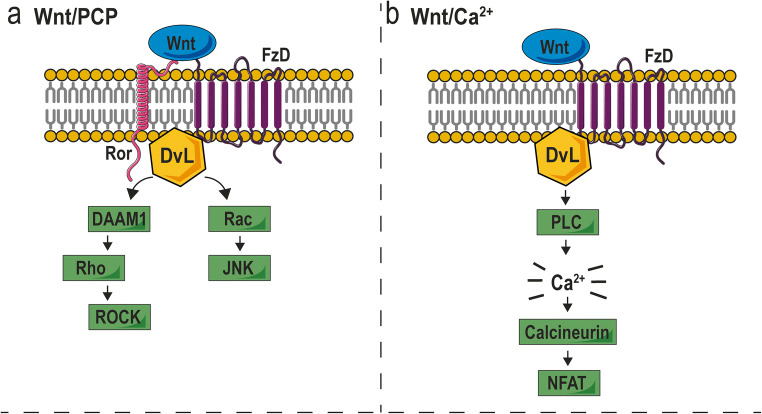
SFRP2 and noncanonical Wnt signaling. **a** The Wnt/PCP pathway is characterized by the recruitment of DvL upon Wnt binding. *Via* two parallel signaling pathways, ROCK and JNK become activated. **b** In the Wnt/Ca^2+^ pathway, a cascade *via* several molecules eventually leads to the release of calcium from the endoplasmic reticulum. In turn, this activates calcineurin to dephosphorylate NFAT and regulates gene transcription. This figure was prepared using Servier Medical Art under a Creative Commons Attribution 3.0 Unported License

In contrast to the canonical Wnt signaling pathway, the noncanonical pathways are independent of β-catenin and can be activated by several Wnt ligands, including Wnt5a, Wnt5B, and Wnt16 [17]. Noncanonical Wnt signaling is divided in two different pathways: the noncanonical planar cell polarity (PCP) pathway and the Wnt/Ca^2+^ pathway. In the PCP pathway, binding of Wnt ligands to the receptor tyrosine kinase–like orphan receptor (Ror)-FzD receptor complex leads to the recruitment of dishevelled (DvL) (Fig. 3a) [18]. Activated DvL can form a complex with the DvL-associated activator of morphogenesis (DAAM)-1, which eventually allows the GTPase activity of Rho to activate the Rho-associated protein kinase (ROCK). In parallel, DvL can also signal *via* the small Rac GTPase, leading to c-Jun N-terminal protein kinase (JNK) activation. This signaling cascade leads to cytoskeletal rearrangements and changes in cell motility. Brinkmann et al. investigated the effect of SFRP2 on the Wnt/PCP pathway [32]. Firstly, they showed that Ror2 and SFPR2 can form a complex in medium of HEK293T cells. Next, HEK293T cells were co-transfected with SFRP2, Ror2, or both, and cells were stimulated with Wnt5a. When both SFRP2 and Ror2 were present, more Wnt5a co-precipitated with Ror2. They finally conclude that SFRP2 stabilizes the Wnt5a-Ror2 complex, activating downstream signaling *via* the Ror2 receptor, enhancing cell movements during gastrulation [32]. The agonistic effect of SFRP2 on the Wnt/PCP pathway is also shown in a totally different cellular context, namely dopamine neurons [33]. In immortalized neuronal SN4741 cells, a low concentration of SFRP2 was able to increase Rac1 activity and promote neuron differentiation.

The second type of noncanonical Wnt signaling, the Wnt/Ca^2+^ pathway, is also initiated upon binding of Wnt ligands to FzD receptors (Fig. 3b). Receptor binding triggers the activation of phospholipase C (PLC), allowing the hydrolyzation of PIP_2_ into diacylglycerol (DAG) and IP_3_ [34]. IP_3_ initiates the release of intracellular calcium from the endoplasmic reticulum, activating several calcium-dependent signaling molecules such as calcineurin. Finally, calcineurin dephosphorylates the nuclear factor of activated T cells (NFAT) which then translocates to the nucleus and regulates gene expression. The Wnt/Ca^2+^ pathway can enhance not only cell proliferation, but also inflammation and metastasis formation in cancer. When murine 2H11 endothelial cells were treated with recombinant SFRP2, a substantial increase in nuclear NFATc3 levels and intracellular calcium influx was observed [35]. These effects were diminished when FzD5 expression was silenced, suggesting a critical role for FzD5 on the activation of the Wnt/Ca^2+^ pathway by SFRP2.

## The functional role of SFRP2 in embryonic development

Several research groups have developed *Sfrp2*^−/−^ homozygous mutant mice to study the function of secreted frizzled-related protein 2 in embryogenesis. Satoh et al. showed that *Sfrp2*^−/−^ mutants have a normal and healthy phenotype. However, in a small percentage of mice, hindlimb syndactyly occurred, meaning that two or more digits are fused together [1]. Morello et al. confirmed the observation that *Sfrp2*^−/−^ mice are viable, fertile, and have a normal lifespan compared to WT mice [23]. However, again, skeletal defects were observed, including shortening of their extremities and toes, also known as brachydactyly. In addition, *Sfrp2* mutants show a kinked tail deformity, which was variable ranging from a small bend to a strong twist in the tail [36].

Deletion of *Sfrp1 *was not lethal either, but when both the *Sfrp1 *and *Sfrp2 *genes were deleted, no pups could be recovered due to pre-natal lethality. These pups died around E16.5, and had defects in limb outgrowth and showed extra digits [1]. This indicates that both proteins are functionally redundant in embryonic development. This redundancy is further confirmed by the fact that expression of SFPR1 is slightly upregulated in the distal limbs of *Sfrp2 *mutant mice [36]. The formation and proper closure of the neural tube are critical during embryonic development of the central nervous system. In *Sfrp*1^−/−^*Sfrp2*^−/−^ mice, neural tube defects were detected from E10.5, including the enlargement of the dorsal neural tube, likely contributing to the observed pre-natal death [37]. These defects could only be observed if both Sfrp genes were completely deleted, indicating their importance in neural tube closure. In another study, *Sfrp1*^−/−^*Sfrp2*^−/−^ mice show a disturbed sexual development, as observed by smaller testes and an abnormal location in de abdominal cavity, more closely located to the kidneys [38]. In females, ovaries are misshaped and are positioned abnormally, as compared to control littermates [38].

## The functional role of SFRP2 in cancer

Overactivation of the Wnt signaling pathway is linked to cancer initiation, progression, and metastasis formation. For example, active Wnt signaling can provide cells with a growth advantage and suppress their differentiation process [39]. In colorectal cancer (CRC), the majority of cases present with overactivation of the canonical Wnt signaling pathway caused by mutations of, e.g., adenomatous polyposis coli (APC) or β-catenin [39]. Mutations in β-catenin are also often observed in many other cancer types, such as hepatocellular carcinoma (HCC), gastric carcinoma, ovarian carcinoma, and melanoma [39, 40]. These gain-of-function mutations disrupt phosphorylation sites and make β-catenin often refractory to proteasomal degradation. On the other hand, mutations of *β-catenin* or *APC* are uncommon in lung cancer [41]. Hyperactivation of the Wnt signaling pathway in this type of cancer is a result of overexpression of DvL proteins or downregulation of Wnt antagonists, eventually leading to an increase of β-catenin. Noncanonical pathways are also likely to be involved in tumorigenesis. The noncanonical PCP and Wnt/Ca^2+^ signaling pathways are involved in cell motility and cell proliferation, respectively. However, there is a high need for further research in this field to fill in the large information gaps that currently exist [41].

As suggested earlier, the effect of SFRP2 on Wnt signaling seems to be context dependent. In cervical cancer cell lines, overexpression of SFRP2 was found to decrease nuclear β-catenin levels, and consequently downregulated gene expression of the cell cycle regulators *C-myc* and *Cyclin D1* [42]. Similarly, overexpression of SFRP2 in oral squamous cell carcinoma cells leads to a downregulation of C*yclin D1* expression [43]. Using a different approach, the treatment of melanoma cells with recombinant SFRP2 also inhibited the expression of β-catenin [44]. Further investigations are needed to see whether a difference in Wnt receptor expression can be observed. Nevertheless, being a key player in the Wnt signaling pathway, SFRP2 is able to influence several branches of tumorigenesis. However, evidence is quite contradictory, describing both tumor promoting and suppressive roles.

### SFRP2 as a tumor suppressor

Many studies have investigated SFRP2 downregulation by promotor hypermethylation in several types of cancer. The *SFRP2* promotor has been described to be (hyper)methylated in bladder cancer [45], breast cancer [46], cervical cancer [47], CRC [48], esophageal cancer [49], gallbladder cancer [50], gastric cancer [51], HCC [52], lung cancer [53], ovarian cancer [54], pancreatic cancer [55], prostate cancer [56], endometrial cancer [57], osteosarcoma [58], oral carcinoma [43], skin cancer [59], and brain tumors [60].

Similarly, SFRP2 mRNA was decreased in osteosarcoma cell lines compared to primary osteoblast cells [58]. A reduced expression has also been observed in pituitary adenoma [61], choriocarcinoma [62], non-small-cell lung carcinoma [63], and glioblastoma [64] compared to their healthy counterparts. When comparing subgroups within the same cancer type, expression of SFRP2 was found to be lower in high grade-, as compared to low grade glioma [64, 65]. SFRP2 expression also seems to be involved in tumor aggressiveness and invasiveness, indicated by the largest SFRP2 downregulation in aggressive [66] and invasive [61] pituitary adenoma compared to their less aggressive or invasive tumor types, respectively.

The fact that SFRP2 is found to be downregulated in a large number of tumor types suggests a tumor suppressor role of the glycoprotein [67]. Indeed, low expression of SFRP2 was associated with a poor clinical outcome in glioblastoma patients [64].

The relationship between SFRP2 expression and tumor growth was further explored in murine tumor models. When nude mice were subcutaneously inoculated with oral squamous cell carcinoma cells or gastric cancer cells overexpressing SFRP2, tumor size was greatly reduced compared to control cells [43, 51]. In an orthotopic model of glioblastoma, overexpression of SFRP2 was also associated with reduced tumor growth and prolonged survival of mice [64]. In a reversed approach, when SFRP2 expression was silenced in choriocarcinoma cells, subcutaneous xenografts grew significantly larger compared to those from cells expressing SFRP2 [62]. Together, these data provide evidence that SFRP2 can function as a tumor suppressor.

### SFRP2 as a tumor promotor

On the contrary, (over)expression of SFRP2 in cancer cell lines and tumor tissues has also been described. Canine mammary tumor cell lines have an abundant SFRP2 expression, while this was not observed in normal mammary gland cells [68]. Similarly, *SFRP2* expression was significantly higher in osteosarcoma tumors compared to mesenchymal stem cells [69]. In bone marrow samples from multiple myeloma patients, SFRP2 could be detected in 10/14 specimens, while only 1/5 bone marrow samples from patients without bone lesions scored positive for SFRP2 expression [70]. When investigating the levels of SFRP2 in serum of breast cancer patients [71], levels were found to be elevated in patients, as compared to controls. High levels of SFRP2 in serum were associated with a poor prognosis. So, in the context of breast cancer, SFRP2 levels in serum may be a promising biomarker and prognostic prediction tool. The relationship between SFRP2 expression and poor survival in breast cancer was further confirmed by Hill et al. [72] and Mohammed et al. [73]*.* A similar correlation between protein expression and prognosis was observed in osteosarcoma [74] and CRC patients [75]. This further confirms that SFRP2 can play a role as tumor promotor.

Direct proof for the tumor promoting effect of SFRP2 can be obtained from *in vivo* tumor mouse models. Yamamura et al. transfected renal carcinoma cells with SFRP2 and monitored their tumor growth potential in nude mice [76]. SFRP2-overexpressing cells generated significantly larger tumors compared to regular renal carcinoma cells, consistent with activated Wnt signaling. Similarly, glioma cells that were experimentally designed to overexpress SFRP2 did generate larger xenografts in athymic mice compared to their non-mutated counterparts [77]. Switching to another approach, treatment of angiosarcoma or breast cancer, using an anti-SFRP2 antibody, results in a significant tumor growth inhibition [78, 79]. Treated tumors displayed no differences in proliferation, but apoptosis was greatly enhanced, as compared to control tumors [79]. Even though this study did not investigate the effect on the Wnt signaling pathway *in vivo*, their *in vitro* data suggest that SFRP2 antagonism results in a reduced level of nuclear β-catenin, unmasking SFRP2 as a Wnt agonist in this setting [78]. Xiao et al. did confirm this agonistic effect on Wnt signaling in lung cancer cell lines [80].

### SFRP2 in metastasis formation

As mentioned earlier, Wnt signaling can also play a major role in the formation of metastasis. Indeed, in late stage cancers, Wnt5a is often upregulated and is known to promote invasion and metastasis formation in breast cancer, melanoma, and gastric cancer [81]. This highlights the role of the noncanonical PCP signaling pathway in this process. Similar effects have been described for the noncanonical Wnt/Ca^2+^ signaling pathway in the context of melanoma [82].

Specifically looking at SFRP2, Techavichit et al. compared the expression levels in both cell lines and tissues samples of metastatic and non-metastatic osteosarcoma, and hit upon a significantly higher SFRP2 expression in metastatic tumors [69]. While overexpression of SFRP2 in osteosarcoma cells did not significantly affect primary tumor growth, a larger number of lung metastases occurred [69]. Similar pro-metastatic effects of SFRP2 were also observed for breast cancer cells [83] and melanoma cells [44]. These data indicate that SFRP2 is a potent stimulator of cell migration and invasion. Montagner et al. investigated which mechanism was responsible for the pro-metastatic effect in melanoma and did not see any effect on canonical Wnt signaling when cells were depleted of SFRP2 [83].

## SFRP2 is an activator of tumor angiogenesis

The generation of a tumor vasculature is a crucial process in tumor progression, providing tumor cells with nutrients and oxygen [84]. However, the newly developed blood vessels in the tumor microenvironment also provide a route for dissemination of cancer cells and, subsequently, metastasis formation [85]. Indeed, it has been shown that the microvessel density within a tumor correlates with its metastatic potential [86]. Furthermore, tumor angiogensis is also involved in the further outgrowth of metastases [87].

The Wnt signaling cascade promotes this process of tumor angiogenesis and endothelial cell survival [88, 89]. Enhanced active β-catenin levels in tumor cells lead to the overexpression of vascular endothelial growth factor (VEGF), an important pro-angiogenic factor, stimulating blood vessel formation [90, 91]. In addition, proteolytic matrix metalloproteinase enzymes (MMPs) are upregulated by canonical Wnt signaling, leading to extracellular matrix degradation during blood vessel formation [92]. The β-catenin-independent Wnt/PCP and Wnt/Ca^2+^ signaling pathways have also been linked to tumor angiogenesis. Defects in the PCP pathway lead to disrupted cell growth and migration of endothelial cells [81], while the Ca^2+^-dependent pathway is involved in the proliferation of endothelial cells and subsequent capillary formation [92, 93].

Several research groups have investigated the upregulation of specific proteins on the tumor vasculature, which might function as promising therapeutic targets in anti-cancer therapies [8, 94–97]. In the vasculature of breast tumors, a significant increase in SFRP2 expression was observed compared to normal breast tissue [5]. Using an anti-SFRP2 antibody, a clear vessel staining was also observed in tissue sections of angiosarcoma, prostate cancer, HCC, CRC, renal cell carcinoma, lung cancer, ovarian cancer, and pancreatic cancer [6, 98]. Indeed, treatment of mice with an anti-SFRP2 antibody shows specific antibody binding to the tumor vasculature but not normal vessels [6, 78].

The upregulation of SFRP2 in the tumor vasculature suggests a link between SFRP2 and tumor angiogenesis. Courtwright et al. were the first to describe a pro-angiogenic effect of SFRP2 [98]. Using a chick chorioallantoic membrane (CAM) assay [99], they observed a large increase in the number of branch points and tube length when the membrane was exposed to SFRP2. In a broad range of *in vitro* experiments, they also showed a beneficial effect on endothelial cell survival and migration. At the genome level, treatment of endothelial cells with SFRP2 upregulates several pro-angiogenic genes such as *VEGF-C* and *cysteine-rich angiogenic inducer (CYR)-61* [74]. Pro-angiogenic data were also be obtained from *in vivo* mouse models, in which melanoma tumors treated with recombinant SFRP2 showed enhanced angiogenesis, which could be reversed by the addition of an anti-SFRP2 antibody [44].

**Fig. 4:**
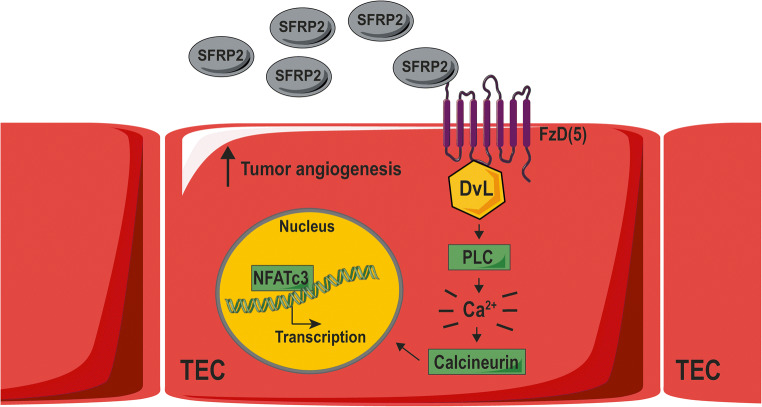
SFRP2 overexpression in tumor endothelium leads to enhanced tumor angiogenesis *via* the Wnt/Ca2^+^ signaling pathway. SFRP2 is overexpressed on tumor endothelial cells (TEC) in several types of cancer. SFRP2 can directly bind to an FzD5 receptor, activating the Wnt/Ca^2+^ pathway. Eventually, the transcription factor NFATc3 will activate several gene transcription events, leading to enhanced tumor angiogenesis. This figure was prepared using Servier Medical Art under a Creative Commons Attribution 3.0 Unported License

Interestingly, SFRP2-treated endothelial cells do also exhibit increased nuclear NFATc3 levels [98, 100], while treatment of endothelial cells with an anti-SFPR2 antibody blocks NFATc3 activation [78]. These data provide evidence that the Wnt/Ca^2+^ pathway plays an important role in the pro-angiogenic effect of SFRP2. This concept was further investigated by Peterson et al.*,* showing that the FzD5 receptor is crucial for SFRP2 mediated Wnt/Ca^2+^ signaling [35]. Endothelial cells lacking this receptor showed reduced intracellular calcium release, no nuclear NFATc3 accumulation, and reduced tube formation upon SFRP2 stimulation. In summary, the pro-angiogenic effect of SFPR2 is largely dependent on noncanonical Wnt/Ca^2+^ signaling, likely *via* direct binding to the FzD5 receptor on tumor endothelial cells (Fig. 4).

## SFRP2 as a therapeutic target in tumor angiogenesis

The secreted glycoprotein SFRP2 is known to regulate Wnt signaling, both *via* the canonical and noncanonical pathways. It is therefore not unexpected that this protein plays important roles in embryonic development and cancer initiation and metastasis formation. A major recent finding is the upregulation of SFRP2 in the tumor vasculature, suggesting it being a specific marker of tumor endothelial cells [5]. The upregulation of SFRP2 can enhance noncanonical Wnt/Ca^2+^ signaling, resulting in enhanced tumor angiogenesis, a crucial step in tumorigenesis [87]. Therefore, targeting of SFRP2 with anti-SFRP2 antibodies or small molecules can disrupt this process, making it a promising approach in anti-cancer therapy.

However, besides upregulation on the tumor vessels, it is important that target candidates are absent or only limitedly expressed on regular blood vessels or other tissues to prevent toxicity. The studies of Fontenot [78] and Garcia [79] provide valuable information on the potential use of anti-SFRP2 antibodies to reduce tumor growth and tumor angiogenesis. Importantly, they did not observe any weight loss or pathological abnormalities in mice treated with these antibodies, suggesting that targeting SFRP2 is a safe anti-cancer strategy. Likewise, molecular imaging in mice using a SFRP2-targeted contrast agent showed specific imaging of the tumor vessels which enhanced by increasing tumor size [6]. This provides evidence that SFRP2 expression is specific for blood vessels in the tumor.

## Conclusion

The glycoprotein SFRP2 is shown to be a key player in the process of tumor angiogensis, an important process in tumor formation and progression. This tumor promoting effect can likely be contributed to the upregulation SFRP2 on the tumor vasculature and, consequently, activation of the noncanonical Wnt/Ca^2+^ pathway. We believe that vaccination against specific tumor endothelial markers is a promising approach to treat or even prevent cancer [101]. Due to its specific expression on the tumor vasculature and the absence of toxicity when treating mice with anti-SFPR2 antibodies, we propose SFRP2 might be a valuable target for vaccination. We previously showed that vaccination against tumor endothelial cell markers leads to the production of target specific antibodies, which are efficient in reducing tumor growth and tumor vessel density in mouse models [9, 101, 102]. Future studies are needed to confirm the potential anti-angiogenic effect of vaccination against SFRP2 in solid tumors.

## Abbreviations

APC: Adenomatous polyposis coli
CAM: Chick chorioallantoic membrane
CRC: Colorectal cancer
CRD: Cysteine-rich domain
CYR: Cysteine-rich angiogenic inducer
DAAM: Dishevelled-associated activator of morphogenesis
DAG: Diacylglycerol
DvL: Dishevelled protein
Fz: Frizzled-like
FzD: Frizzled
HCC: Hepatocellular carcinoma
JNK: c-Jun N-terminal protein kinase
LRP: Low-density lipoprotein receptor–related protein
MMPs: Matrix metalloproteinases
NFAT: Nuclear factor of activated T cells
NTR: Netrin domain
PCP: Planar cell polarity
PLC: Phospholipase C
ROCK: Rho-associated protein kinase
Ror: Receptor tyrosine kinase–like orphan receptor
SARP: Secreted apoptosis-related protein
SDF: Stromal cell–derived factor
SFRP: Secreted frizzled-related protein
TEC: Tumor endothelial cell
VEGF: Vascular endothelial growth factor
Wnt: Wingless-related integration site
β-TrCp: F-box β-transducing repeat-containing protein

## References

[1] Satoh W, Gotoh T, Tsunematsu Y, Aizawa S, Shimono A (2006). Sfrp1 and Sfrp2 regulate anteroposterior axis elongation and somite segmentation during mouse embryogenesis. Development.

[2] Banyai L, Patthy L (1999). The NTR module: domains of netrins, secreted frizzled related proteins, and type I procollagen C-proteinase enhancer protein are homologous with tissue inhibitors of metalloproteases. Protein Science.

[3] Finch PW, He X, Kelley MJ, Uren A, Schaudies RP, Popescu NC, Rudikoff S, Aaronson SA, Varmus HE, Rubin JS (1997). Purification and molecular cloning of a secreted, frizzled-related antagonist of Wnt action. Proceedings of the National Academy of Sciences of the United States of America.

[4] Jung YS, Park JI (2020). Wnt signaling in cancer: therapeutic targeting of Wnt signaling beyond β-catenin and the destruction complex. Experimental & Molecular Medicine.

[5] Bhati R, Patterson C, Livasy CA, Fan C, Ketelsen D, Hu Z, Reynolds E, Tanner C, Moore DT, Gabrielli F, Perou CM, Klauber-DeMore N (2008). Molecular characterization of human breast tumor vascular cells. The American Journal of Pathology.

[6] Tsuruta JK, Klauber-DeMore N, Streeter J, Samples J, Patterson C, Mumper RJ, Ketelsen D, Dayton P (2014). Ultrasound molecular imaging of secreted frizzled related protein-2 expression in murine angiosarcoma. PLoS One.

[7] Ramjiawan RR, Griffioen AW, Duda DG (2017). Anti-angiogenesis for cancer revisited: Is there a role for combinations with immunotherapy?. Angiogenesis.

[8] van Loon, K., Yemelyanenko-Lyalenko, J., Margadant, C., Griffioen, A. W., & Huijbers, E. J. M. (2020). Role of fibrillin-2 in the control of TGF-β activation in tumor angiogenesis and connective tissue disorders. *Biochimica Et Biophysica Acta. Reviews on Cancer, 1873*(2), 188354. 10.1016/j.bbcan.2020.188354. 10.1016/j.bbcan.2020.188354 32119940

[9] Huijbers EJM, van der Werf IM, Faber LD, Sialino LD, van der Laan P, Holland HA, Cimpean AM, Thijssen VLJL, van Beijnum JR, Griffioen AW (2019). Targeting tumor vascular CD99 inhibits tumor growth. Frontiers in Immunology.

[10] Zhan T, Rindtorff N, Boutros M (2017). Wnt signaling in cancer. Oncogene.

[11] Sharma R (1973). Wingless, a new mutant in D. melanogaster. Drosophila Information Service.

[12] Nusse R, Varmus HE (1982). Many tumors induced by the mouse mammary tumor virus contain a provirus integrated in the same region of the host genome. Cell.

[13] Rijsewijk F, Schuermann M, Wagenaar E, Parren P, Weigel D, Nusse R (1987). The Drosophila homolog of the mouse mammary oncogene int-1 is identical to the segment polarity gene wingless. Cell.

[14] Shirozu M, Tada H, Tashiro K, Nakamura T, Lopez ND, Nazarea M, Hamada T, Sato T, Nakano T, Honjo T (1996). Characterization of novel secreted and membrane proteins isolated by the signal sequence trap method. Genomics.

[15] Melkonyan HS, Chang WC, Shapiro JP, Mahadevappa M, Fitzpatrick PA, Kiefer MC, Tomei LD, Umansky SR (1997). SARPs: a family of secreted apoptosis-related proteins. Proceedings of the National Academy of Sciences of the United States of America.

[16] Katoh M, Katoh M (2005). Comparative genomics on SFRP2 orthologs. Oncology Reports.

[17] Benhaj K, Akcali KC, Ozturk M (2006). Redundant expression of canonical Wnt ligands in human breast cancer cell lines. Oncology Reports.

[18] Wu Y, Liu X, Zheng H, Zhu H, Mai W, Huang X, Huang Y (2020). Multiple roles of sFRP2 in cardiac development and cardiovascular disease. International Journal of Biological Sciences.

[19] Alfaro MP, Vincent A, Saraswati S, Thorne CA, Hong CC, Lee E, Young PP (2010). sFRP2 suppression of bone morphogenic protein (BMP) and Wnt signaling mediates mesenchymal stem cell (MSC) self-renewal promoting engraftment and myocardial repair. The Journal of Biological Chemistry.

[20] Wawrzak D, Metioui M, Willems E, Hendrickx M, de Genst E, Leyns L (2007). Wnt3a binds to several sFRPs in the nanomolar range. Biochemical and Biophysical Research Communications.

[21] Hua Y, Yang Y, Li Q, He X, Zhu W, Wang J, Gan X (2018). Oligomerization of frizzled and LRP5/6 protein initiates intracellular signaling for the canonical WNT/beta-catenin pathway. The Journal of Biological Chemistry.

[22] Galli LM, Barnes T, Cheng T, Acosta L, Anglade A, Willert K, Nusse R, Burrus LW (2006). Differential inhibition of Wnt-3a by Sfrp-1, Sfrp-2, and Sfrp-3. Developmental Dynamics.

[23] Morello R, Bertin TK, Schlaubitz S, Shaw CA, Kakuru S, Munivez E, Hermanns P, Chen Y, Zabel B, Lee B (2008). Brachy-syndactyly caused by loss of Sfrp2 function. Journal of Cellular Physiology.

[24] Zhang Z, Deb A, Zhang Z, Pachori A, He W, Guo J, Pratt R, Dzau VJ (2009). Secreted frizzled related protein 2 protects cells from apoptosis by blocking the effect of canonical Wnt3a. Journal of Molecular and Cellular Cardiology.

[25] Kress E, Rezza A, Nadjar J, Samarut J, Plateroti M (2009). The frizzled-related sFRP2 gene is a target of thyroid hormone receptor alpha1 and activates beta-catenin signaling in mouse intestine. The Journal of Biological Chemistry.

[26] Mastri M, Shah Z, Hsieh K, Wang X, Wooldridge B, Martin S, Suzuki G, Lee T (2014). Secreted frizzled-related protein 2 as a target in antifibrotic therapeutic intervention. American Journal of Physiology. Cell Physiology.

[27] Lin M, Liu X, Zheng H, Huang X, Wu Y, Huang A, Zhu H, Hu Y, Mai W, Huang Y (2020). IGF-1 enhances BMSC viability, migration, and anti-apoptosis in myocardial infarction via secreted frizzled-related protein 2 pathway. Stem Cell Research & Therapy.

[28] Heinosalo T, Gabriel M, Kallio L, Adhikari P, Huhtinen K, Laajala TD, Kaikkonen E, Mehmood A, Suvitie P, Kujari H, Aittokallio T, Perheentupa A, Poutanen M (2018). Secreted frizzled-related protein 2 (SFRP2) expression promotes lesion proliferation via canonical WNT signaling and indicates lesion borders in extraovarian endometriosis. Human Reproduction.

[29] von Marschall Z, Fisher LW (2010). Secreted frizzled-related protein-2 (sFRP2) augments canonical Wnt3a-induced signaling. Biochemical and Biophysical Research Communications.

[30] Skah S, Nadjar J, Sirakov M, Plateroti M (2015). The secreted frizzled-related protein 2 modulates cell fate and the Wnt pathway in the murine intestinal epithelium. Experimental Cell Research.

[31] Xavier CP, Melikova M, Chuman Y, Uren A, Baljinnyam B, Rubin JS (2014). Secreted frizzled-related protein potentiation versus inhibition of Wnt3a/beta-catenin signaling. Cellular Signalling.

[32] Brinkmann EM, Mattes B, Kumar R, Hagemann AI, Gradl D, Scholpp S (2016). Secreted frizzled-related protein 2 (sFRP2) redirects non-canonical Wnt signaling from Fz7 to Ror2 during vertebrate gastrulation. The Journal of Biological Chemistry.

[33] Kele J, Andersson ER, Villaescusa JC, Cajanek L, Parish CL, Bonilla S, Toledo EM, Bryja V, Rubin JS, Shimono A, Arenas E (2012). SFRP1 and SFRP2 dose-dependently regulate midbrain dopamine neuron development in vivo and in embryonic stem cells. Stem Cells.

[34] Qin JJ, Nag S, Wang W, Zhou J, Zhang WD, Wang H, Zhang R (2014). NFAT as cancer target: mission possible?. Biochimica et Biophysica Acta.

[35] Peterson YK, Nasarre P, Bonilla IV, Hilliard E, Samples J, Morinelli TA, Hill EG, Klauber-DeMore N (2017). Frizzled-5: a high affinity receptor for secreted frizzled-related protein-2 activation of nuclear factor of activated T-cells c3 signaling to promote angiogenesis. Angiogenesis.

[36] Ikegawa M, Han H, Okamoto A, Matsui R, Tanaka M, Omi N, Miyamae M, Toguchida J, Tashiro K (2008). Syndactyly and preaxial synpolydactyly in the single Sfrp2 deleted mutant mice. Developmental Dynamics.

[37] Misra K, Matise MP (2010). A critical role for sFRP proteins in maintaining caudal neural tube closure in mice via inhibition of BMP signaling. Developmental Biology.

[38] Warr N, Siggers P, Bogani D, Brixey R, Pastorelli L, Yates L, Dean CH, Wells S, Satoh W, Shimono A, Greenfield A (2009). Sfrp1 and Sfrp2 are required for normal male sexual development in mice. Developmental Biology.

[39] Giles RH, van Es JH, Clevers H (2003). Caught up in a Wnt storm: Wnt signaling in cancer. Biochimica et Biophysica Acta.

[40] Howe LR, Brown AM (2004). Wnt signaling and breast cancer. Cancer Biology & Therapy.

[41] Mazieres J, He B, You L, Xu Z, Jablons DM (2005). Wnt signaling in lung cancer. Cancer Letters.

[42] Chung MT, Lai HC, Sytwu HK, Yan MD, Shih YL, Chang CC, Yu MH, Liu HS, Chu DW, Lin YW (2009). SFRP1 and SFRP2 suppress the transformation and invasion abilities of cervical cancer cells through Wnt signal pathway. Gynecologic Oncology.

[43] Xiao C, Wang L, Zhu L, Zhang C, Zhou J (2014). Secreted frizzledrelated protein 2 is epigenetically silenced and functions as a tumor suppressor in oral squamous cell carcinoma. Molecular Medicine Reports.

[44] Kaur A, Webster MR, Marchbank K, Behera R, Ndoye A, Kugel CH (2016). sFRP2 in the aged microenvironment drives melanoma metastasis and therapy resistance. Nature.

[45] Marsit CJ, Karagas MR, Andrew A, Liu M, Danaee H, Schned AR, Nelson HH, Kelsey KT (2005). Epigenetic inactivation of SFRP genes and TP53 alteration act jointly as markers of invasive bladder cancer. Cancer Research.

[46] Li Z, Guo X, Wu Y, Li S, Yan J, Peng L, Xiao Z, Wang S, Deng Z, Dai L, Yi W, Xia K, Tang L, Wang J (2015). Methylation profiling of 48 candidate genes in tumor and matched normal tissues from breast cancer patients. Breast Cancer Research and Treatment.

[47] Lin YW, Chung MT, Lai HC, De Yan M, Shih YL, Chang CC (2009). Methylation analysis of SFRP genes family in cervical adenocarcinoma. Journal of Cancer Research and Clinical Oncology.

[48] Liu X, Fu J, Bi H, Ge A, Xia T, Liu Y, Sun H, Li D, Zhao Y (2019). DNA methylation of SFRP1, SFRP2, and WIF1 and prognosis of postoperative colorectal cancer patients. BMC Cancer.

[49] Zou H, Molina JR, Harrington JJ, Osborn NK, Klatt KK, Romero Y, Burgart LJ, Ahlquist DA (2005). Aberrant methylation of secreted frizzled-related protein genes in esophageal adenocarcinoma and Barrett’s esophagus. International Journal of Cancer.

[50] Zhang Y, Yang B, Du Z, Gao YT, Wang YJ, Jing X (2010). Identification and validation of specific methylation profile in bile for differential diagnosis of malignant biliary stricture. Clinical Biochemistry.

[51] Cheng YY, Yu J, Wong YP, Man EP, Jin VX, To, K. F (2007). Frequent epigenetic inactivation of secreted frizzled-related protein 2 (SFRP2) by promoter methylation in human gastric cancer. British Journal of Cancer.

[52] Takagi H, Sasaki S, Suzuki H, Toyota M, Maruyama R, Nojima M, Yamamoto H, Omata M, Tokino T, Imai K, Shinomura Y (2008). Frequent epigenetic inactivation of SFRP genes in hepatocellular carcinoma. Journal of Gastroenterology.

[53] Liu S, Chen X, Chen R, Wang J, Zhu G, Jiang J (2017). Diagnostic role of Wnt pathway gene promoter methylation in non small cell lung cancer. Oncotarget.

[54] Su HY, Lai HC, Lin YW, Chou YC, Liu CY, Yu MH (2009). An epigenetic marker panel for screening and prognostic prediction of ovarian cancer. International Journal of Cancer.

[55] Bu XM, Zhao CH, Zhang N, Gao F, Lin S, Dai XW (2008). Hypermethylation and aberrant expression of secreted frizzled-related protein genes in pancreatic cancer. World Journal of Gastroenterology.

[56] Perry AS, O’Hurley G, Raheem OA, Brennan K, Wong S, O’Grady A, Kennedy AM, Marignol L, Murphy TM, Sullivan L, Barrett C, Loftus B, Thornhill J, Hewitt SM, Lawler M, Kay E, Lynch T, Hollywood D (2013). Gene expression and epigenetic discovery screen reveal methylation of SFRP2 in prostate cancer. International Journal of Cancer.

[57] Suehiro Y, Okada T, Okada T, Anno K, Okayama N, Ueno K, Hiura M, Nakamura M, Kondo T, Oga A, Kawauchi S, Hirabayashi K, Numa F, Ito T, Saito T, Sasaki K, Hinoda Y (2008). Aneuploidy predicts outcome in patients with endometrial carcinoma and is related to lack of CDH13 hypermethylation. Clinical Cancer Research.

[58] Xiao Q, Yang Y, Zhang X, An Q (2016). Enhanced Wnt signaling by methylation-mediated loss of SFRP2 promotes osteosarcoma cell invasion. Tumour Biology.

[59] Liang J, Kang X, Halifu Y, Zeng X, Jin T, Zhang M, Luo D, Ding Y, Zhou Y, Yakeya B, Abudu D, Pu X (2015). Secreted frizzled-related protein promotors are hypermethylated in cutaneous squamous carcinoma compared with normal epidermis. BMC Cancer.

[60] Gotze S, Wolter M, Reifenberger G, Muller O, Sievers S (2010). Frequent promoter hypermethylation of Wnt pathway inhibitor genes in malignant astrocytic gliomas. International Journal of Cancer.

[61] Ren J, Jian F, Jiang H, Sun Y, Pan S, Gu C, Chen X, Wang W, Ning G, Bian L, Sun Q (2018). Decreased expression of SFRP2 promotes development of the pituitary corticotroph adenoma by upregulating Wnt signaling. International Journal of Oncology.

[62] Zeng X, Zhang Y, Xu H, Zhang T, Xue Y, An R (2018). Secreted frizzled related protein 2 modulates epithelial-mesenchymal transition and Stemness via Wnt/beta-catenin signaling in choriocarcinoma. Cellular Physiology and Biochemistry.

[63] Li P, Zhao S, Hu Y (2019). SFRP2 modulates nonsmall cell lung cancer A549 cell apoptosis and metastasis by regulating mitochondrial fission via Wnt pathways. Molecular Medicine Reports.

[64] Han M, Wang S, Fritah S, Wang X, Zhou W, Yang N, Ni S, Huang B, Chen A, Li G, Miletic H, Thorsen F, Bjerkvig R, Li X, Wang J (2020). Interfering with long non-coding RNA MIR22HG processing inhibits glioblastoma progression through suppression of Wnt/beta-catenin signalling. Brain.

[65] Leventoux N, Augustus M, Azar S, Riquier S, Villemin JP, Guelfi S, Falha L, Bauchet L, Gozé C, Ritchie W, Commes T, Duffau H, Rigau V, Hugnot JP (2020). Transformation foci in IDH1-mutated gliomas show STAT3 phosphorylation and downregulate the metabolic enzyme ETNPPL, a negative regulator of glioma growth. Scientific Reports.

[66] Wu Y, Bai J, Hong L, Liu C, Yu S, Yu G (2016). Low expression of secreted frizzled-related protein 2 and nuclear accumulation of beta-catenin in aggressive nonfunctioning pituitary adenoma. Oncology Letters.

[67] Esteve P, Bovolenta P (2010). The advantages and disadvantages of sfrp1 and sfrp2 expression in pathological events. The Tohoku Journal of Experimental Medicine.

[68] Lee JL, Chang CJ, Chueh LL, Lin CT (2003). Expression of secreted frizzled-related protein 2 in a primary canine mammary tumor cell line: A candidate tumor marker for mammary tumor cells. In Vitro Cellular & Developmental Biology. Animal.

[69] Techavichit P, Gao Y, Kurenbekova L, Shuck R, Donehower LA, Yustein JT (2016). Secreted frizzled-related protein 2 (sFRP2) promotes osteosarcoma invasion and metastatic potential. BMC Cancer.

[70] Oshima T, Abe M, Asano J, Hara T, Kitazoe K, Sekimoto E, Tanaka Y, Shibata H, Hashimoto T, Ozaki S, Kido S, Inoue D, Matsumoto T (2005). Myeloma cells suppress bone formation by secreting a soluble Wnt inhibitor, sFRP-2. Blood.

[71] Huang C, Ye Z, Wan J, Liang J, Liu M, Xu X, Li L (2019). Secreted frizzled-related protein 2 is associated with disease progression and poor prognosis in breast cancer. Disease Markers.

[72] Hill VK, Ricketts C, Bieche I, Vacher S, Gentle D, Lewis C, Maher ER, Latif F (2011). Genome-wide DNA methylation profiling of CpG islands in breast cancer identifies novel genes associated with tumorigenicity. Cancer Research.

[73] Mohammed, S. I., Utturkar, S., Lee, M., Yang, H. H., Cui, Z., Atallah Lanman, N., et al. (2020). Ductal carcinoma in situ progression in dog model of breast cancer. *Cancers (Basel), 12*(2). 10.3390/cancers12020418. 10.3390/cancers12020418 PMC7072653 32053966

[74] Kim H, Yoo S, Zhou R, Xu A, Bernitz JM, Yuan Y, Gomes AM, Daniel MG, Su J, Demicco EG, Zhu J, Moore KA, Lee DF, Lemischka IR, Schaniel C (2018). Oncogenic role of SFRP2 in p53-mutant osteosarcoma development via autocrine and paracrine mechanism. Proceedings of the National Academy of Sciences of the United States of America.

[75] Vincent KM, Postovit LM (2017). A pan-cancer analysis of secreted frizzled-related proteins: re-examining their proposed tumour suppressive function. Scientific Reports.

[76] Yamamura S, Kawakami K, Hirata H, Ueno K, Saini S, Majid S, Dahiya R (2010). Oncogenic functions of secreted frizzled-related protein 2 in human renal cancer. Molecular Cancer Therapeutics.

[77] Roth W, Wild-Bode C, Platten M, Grimmel C, Melkonyan HS, Dichgans J, Weller M (2000). Secreted frizzled-related proteins inhibit motility and promote growth of human malignant glioma cells. Oncogene.

[78] Fontenot E, Rossi E, Mumper R, Snyder S, Siamakpour-Reihani S, Ma P, Hilliard E, Bone B, Ketelsen D, Santos C, Patterson C, Klauber-DeMore N (2013). A novel monoclonal antibody to secreted frizzled-related protein 2 inhibits tumor growth. Molecular Cancer Therapeutics.

[79] Garcia D, Nasarre P, Bonilla IV, Hilliard E, Peterson YK, Spruill L, Broome AM, Hill EG, Yustein JT, Mehrotra S, Klauber-DeMore N (2019). Development of a novel humanized monoclonal antibody to secreted frizzled-related protein-2 that inhibits triple-negative breast cancer and angiosarcoma growth in vivo. Annals of Surgical Oncology.

[80] Xiao X, Xiao Y, Wen R, Zhang Y, Li X, Wang H (2015). Promoting roles of the secreted frizzled-related protein 2 as a Wnt agonist in lung cancer cells. Oncology Reports.

[81] Wang Y (2009). Wnt/planar cell polarity signaling: a new paradigm for cancer therapy. Molecular Cancer Therapeutics.

[82] Webster MR, Kugel CH, Weeraratna AT (2015). The Wnts of change: How Wnts regulate phenotype switching in melanoma. Biochimica et Biophysica Acta.

[83] Montagner M, Bhome R, Hooper S, Chakravarty P, Qin X, Sufi J, Bhargava A, Ratcliffe CDH, Naito Y, Pocaterra A, Tape CJ, Sahai E (2020). Crosstalk with lung epithelial cells regulates Sfrp2-mediated latency in breast cancer dissemination. Nature Cell Biology.

[84] Griffioen AW, Molema G (2000). Angiogenesis: potentials for pharmacologic intervention in the treatment of cancer, cardiovascular diseases, and chronic inflammation. Pharmacological Reviews.

[85] Bielenberg DR, Zetter BR (2015). The contribution of angiogenesis to the process of metastasis. Cancer Journal (Sudbury, Mass.).

[86] Kerbel R, Folkman J (2002). Clinical translation of angiogenesis inhibitors. Nature Reviews Cancer.

[87] Hillen F, Griffioen AW (2007). Tumour vascularization: sprouting angiogenesis and beyond. Cancer Metastasis Reviews.

[88] Olsen JJ, Pohl SO, Deshmukh A, Visweswaran M, Ward NC, Arfuso F (2017). The role of Wnt Signalling in angiogenesis. Clinical Biochemist Reviews.

[89] Brandt MM, van Dijk CGM, Chrifi I, Kool HM, Bürgisser PE, Louzao-Martinez L, Pei J, Rottier RJ, Verhaar MC, Duncker DJ, Cheng C (2018). Endothelial loss of Fzd5 stimulates PKC/Ets1-mediated transcription of Angpt2 and Flt1. Angiogenesis.

[90] Danieau, G., Morice, S., Redini, F., Verrecchia, F., & Royer, B. B. (2019). New insights about the Wnt/beta-catenin signaling pathway in primary bone tumors and their microenvironment: a promising target to develop therapeutic strategies? *International Journal of Molecular Sciences, 20*(15). 10.3390/ijms20153751. 10.3390/ijms20153751 PMC6696068 31370265

[91] Easwaran V, Lee SH, Inge L, Guo L, Goldbeck C, Garrett E (2003). Beta-catenin regulates vascular endothelial growth factor expression in colon cancer. Cancer Research.

[92] Rapp J, Jaromi L, Kvell K, Miskei G, Pongracz JE (2017). WNT signaling - lung cancer is no exception. Respiratory Research.

[93] Cirone P, Lin S, Griesbach HL, Zhang Y, Slusarski DC, Crews CM (2008). A role for planar cell polarity signaling in angiogenesis. Angiogenesis.

[94] van Beijnum JR, Dings RP, van der Linden E, Zwaans BM, Ramaekers FC, Mayo KH (2006). Gene expression of tumor angiogenesis dissected: specific targeting of colon cancer angiogenic vasculature. Blood.

[95] St Croix B, Rago C, Velculescu V, Traverso G, Romans KE, Montgomery E (2000). Genes expressed in human tumor endothelium. Science.

[96] van Beijnum JR, Griffioen AW (2005). In silico analysis of angiogenesis associated gene expression identifies angiogenic stage related profiles. Biochimica et Biophysica Acta.

[97] Thijssen VL, Postel R, Brandwijk RJ, Dings RP, Nesmelova I, Satijn S (2006). Galectin-1 is essential in tumor angiogenesis and is a target for antiangiogenesis therapy. Proceedings of the National Academy of Sciences of the United States of America.

[98] Courtwright A, Siamakpour-Reihani S, Arbiser JL, Banet N, Hilliard E, Fried L, Livasy C, Ketelsen D, Nepal DB, Perou CM, Patterson C, Klauber-DeMore N (2009). Secreted frizzle-related protein 2 stimulates angiogenesis via a calcineurin/NFAT signaling pathway. Cancer Research.

[99] Nowak-Sliwinska P, Segura T, Iruela-Arispe ML (2014). The chicken chorioallantoic membrane model in biology, medicine and bioengineering. Angiogenesis.

[100] Siamakpour-Reihani S, Caster J, Bandhu Nepal D, Courtwright A, Hilliard E, Usary J, Ketelsen D, Darr D, Shen XJ, Patterson C, Klauber-DeMore N (2011). The role of calcineurin/NFAT in SFRP2 induced angiogenesis--a rationale for breast cancer treatment with the calcineurin inhibitor tacrolimus. PLoS One.

[101] Huijbers EJ, Ringvall M, Femel J, Kalamajski S, Lukinius A, Abrink M (2010). Vaccination against the extra domain-B of fibronectin as a novel tumor therapy. The FASEB Journal.

[102] Huijbers EJM, van Beijnum JR, Lê CT, Langman S, Nowak-Sliwinska P, Mayo KH, Griffioen AW (2018). An improved conjugate vaccine technology; induction of antibody responses to the tumor vasculature. Vaccine.

